# Human intention recognition by deep LSTM and transformer networks for real-time human-robot collaboration

**DOI:** 10.3389/frobt.2025.1708987

**Published:** 2025-12-19

**Authors:** Matija Mavsar, Mihael Simonič, Aleš Ude

**Affiliations:** Humanoid and Cognitive Robotics Laboratory, Department of Automatics, Biocybernetics, and Robotics, Jožef Stefan Institute, Ljubljana, Slovenia

**Keywords:** human-robot collaboration, deep neural networks, LSTM, transformer, intention recognition

## Abstract

Collaboration between humans and robots is essential for optimizing the performance of complex tasks in industrial environments, reducing worker strain, and improving safety. This paper presents an integrated human-robot collaboration (HRC) system that leverages advanced intention recognition for real-time task sharing and interaction. By utilizing state-of-the-art human pose estimation combined with deep learning models, we developed a robust framework for detecting and predicting worker intentions. Specifically, we employed LSTM-based and transformer-based neural networks with convolutional and pooling layers to classify human hand trajectories, achieving higher accuracy compared to previous approaches. Additionally, our system integrates dynamic movement primitives (DMPs) for smooth robot motion transitions, collision prevention, and automatic motion onset/cessation detection. We validated the system in a real-world industrial assembly task, demonstrating its effectiveness in enhancing the fluency, safety, and efficiency of human-robot collaboration. The proposed method shows promise in improving real-time decision-making in collaborative environments, offering a safer and more intuitive interaction between humans and robots.

## 1 Introduction

The growing availability of collaborative robots in the market has paved the way for the development of human-robot collaboration (HRC) approaches, designed to enhance the efficiency of workspace sharing between humans and robots. The primary goal of HRC is to enable robots to perform tasks that would be too complex to execute independently, while simultaneously alleviating the burden on human workers by delegating the most challenging and repetitive aspects of the work to the robots.

One of the possible ways to increase efficiency of cooperation is through recognition and anticipation of human worker activity. Human pose estimation and prediction are crucial in this context, as they enable robots to better understand and respond to human behavior. By anticipating and classifying human motions, we can predict intentions and adapt the robots’ behavior accordingly. This leads to more natural and intuitive human-robot interactions. For example, if a robot can predict that a human is reaching for an object, it can offer assistance or adjust its own movements to avoid interference.

Machine vision can be employed to obtain useful information about the state of the cooperative workspace and control the robot in a way that increases safety and fluidity of collaboration. Recurrent neural networks (RNNs) are a promising technology for collaborative tasks that require anticipation of an agent’s motion since. One variant are RNNs with long short-term memory (LSTM) (Hochreiter and Schmidhuber, 1997) units, which can analyze time-dependent processes based on partially observed data and predict future states, as opposed to vanilla neural networks, which require entire inputs, e.g., motion trajectories, to provide predictions. RNNs have for example been utilized for labeling or predicting human motion based on measurements of past poses or captured images (Zhang et al., 2020; Wang et al., 2019; Yang et al., 2021; Mavsar et al., 2024). Another deep neural architecture that has seen a big rise in popularity are transformer networks with attention mechanism (Vaswani et al., 2017), which are largely being used for natural language processing tasks, as well as for trajectory prediction (Giuliari et al., 2021). While mostly employed for sequence-to-sequence tasks, they are also used for sequence classification tasks, e.g. trajectory classification. These techniques, along with increasingly capable and affordable sensors such as depth cameras, enable efficient processing of information in a HRC system and, consequently, optimization of the collaborative process.

**FIGURE 1:**
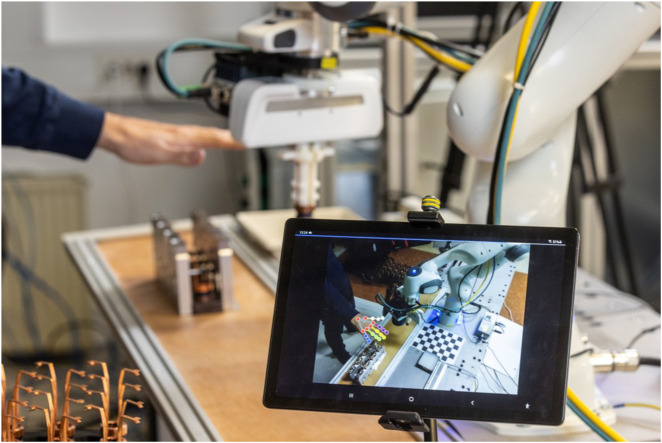
Experimental collaborative setup in a real industrial workcell. Human and robot cooperate in picking up copper rings from the loading area (bottom left part of the image) and placing them into one of the slots inside the casting model on the table. Since they are performing the task simultaneously, we employ an integrated supervision system to detect the worker’s hand and predict their intention, in order to adapt the robot motion accordingly.

In this paper, we present an integrated system for supervision of a collaborative environment that facilitates dynamic and safe task sharing by utilizing a single RGB-D camera (Figure 1). We employ an existing human body pose estimation method and combine it with depth information from the camera to obtain position of the worker’s hand in global coordinates. To increase task fluency, we perform human intention recognition by classifying the observed hand trajectory in real time, where we compare several deep learning architectures for sequence classification. Moreover, we successfully integrated collision prevention and automatic initiation of motion prediction to enhance the autonomous functionality of our system. The first one is ensured by monitoring the distance between the human and the robot, enabling us to identify and avoid potential collisions, and the second one by detecting the onset and cessation of worker’s motion to determine when the intention recognition system should start and stop forecasting the worker’s hand trajectory.

The main contributions of this paper are:

1. Two intention recognition architectures (LSTM- and transformer-based with convolution/pooling layers) that achieve higher real-time classification accuracy than a recent transformer baseline.
2. A third-order quaternion formulation of Dynamic Movement Primitives (DMPs) in Cartesian space, enabling smooth trajectory and orientation switching when goals change.
3. An integrated HRC supervision system that combines intention recognition, motion onset/cessation detection, DMP-based motion generation, and adaptive collision prevention, validated in an industrial assembly scenario for improved fluency and safety.

## 2 Related work

### 2.1 Human-robot collaboration (HRC)

The field of human-robot collaboration (HRC) has seen significant progress in recent years, driven by the increasing demand for service robots in both home and industrial environments (Ajoudani et al., 2018). In such settings, robots must operate seamlessly with humans to accomplish shared tasks. A review by Matheson et al. (2019) highlights the growing use of collaborative robots (cobots) in HRC research. Cobots incorporate features such as force and torque sensors, force limits, and anti-collision systems to enhance safe and effective collaboration. Key research goals in HRC include improving task performance, enabling robot learning through physical interaction (Simonič et al., 2021), and ensuring both fluency (Hoffman, 2019) and safety (Marvel and Norcross, 2017; Byner et al., 2019).

### 2.2 Intention recognition and prediction methods

A crucial step toward achieving fluent collaboration is enabling robots to recognize and predict human intentions. Accurate prediction enhances control efficiency and boosts overall productivity. Spatial-Temporal Graph Convolutional Networks (ST-GCNs) have been proposed for skeleton-based action recognition (Yan et al., 2018), automatically learning both spatial and temporal patterns of human joints. These networks demonstrate strong generalization capabilities without relying on hand-crafted features, although they require complete trajectories before making predictions.

Recurrent neural networks (RNNs) have been applied for activity recognition, such as predicting description labels from RGB-D videos (Wang et al., 2017) or classifying 2D trajectories into travel categories (Liu et al., 2019). Other methods employ skeleton motion data to predict future poses (Zhang et al., 2020; Yasar and Iqbal, 2021) or action probability distributions (Schydlo et al., 2018), while Abuduweili et al. (2019) use RNN with attention, but do not explore the effect of preprocessing. Moon and Seo (2019) combine robot, haptic and depth image data to predict future human positions using an RNN, however do not perform classification. An alternative to RNNs is the framework by Callens et al. (2020), which uses probabilistic principal component analysis to learn motion models. Hybrid methods, combining both learning and model-based optimization for human intention recognition and robot control have also been proposed; Gao et al. (2021) introduced a hybrid recurrent neural network combining improved bidirectional and unidirectional LSTM layers for intention recognition from human motion data, while Tassi et al. (2022) developed a vision-based ergonomic HRC framework to ensure smooth, adaptive, and ergonomic robot motion.

Ali et al. (2024) explore the use of Large Language Models (LLMs) to infer human intentions in a collaborative object categorization task with a physical robot, while Jing et al. (2025) employ LLMs for intention recognition in the context of spacecrafts. Although these works demonstrate the feasibility of intention recognition using Large Language Models (LLMs), our task requires specialized classifiers trained explicitly for continuous hand-trajectory data. Unlike general-purpose LLMs designed for high-level reasoning over textual or multimodal inputs, our system demands a lightweight, domain-specific model capable of real-time inference.

While originally developed for natural language processing, transformer networks have recently been adapted for motion-related tasks. They have shown strong performance in pedestrian intention recognition (Sui et al., 2021), pedestrian trajectory forecasting (Yu et al., 2020; Sui et al., 2021), and trajectory classification (Liang et al., 2022). A simpler architecture with adaptive pooling layers has also proven successful in action recognition tasks (Abdu-Aguye et al., 2020). Moreover, Pettersson and Falkman (2023) compared gaze-based human intention recognition using both LSTM and transformer-based networks. We thus decided to combine the efficacy of pooling layers, transformers and LSTM networks into novel architectures and compare them to baselines without preprocessing.

### 2.3 Dynamic movement primitives

Dynamic Movement Primitives (DMPs) provide smooth, timescalable motion representations for dynamic tasks (Nemec and Ude, 2012). In HRC, DMPs have been adapted to increase adaptability and robustness. Tu et al. (2022) used coupled DMPs to coordinate arm and base motions for compliant whole-body control, while Cai and Liu (2023) introduced a probabilistic DMP framework that removes frame dependency, and Sidiropoulos and Doulgeri (2024) improved spatial generalization with dynamic via-points.

Earlier studies applied DMPs to classical HRC tasks such as handovers and repetitive actions. Prada et al. (2013) showed that DMPs adapt effectively to moving goals in handover scenarios, while Gams et al. (2014) used periodic DMPs for tasks like surface wiping with force feedback and human coaching. Overall, these works highlight the ability of DMPs to adapt trajectories online, though safety and collision avoidance are often handled by separate modules. The integration of motion onset detection or partial trajectory data into DMP frameworks remains underexplored, offering potential for combining predictive intention recognition with adaptive control.

While modern learning-based approaches such as imitation learning and reinforcement learning are powerful (Byeon et al., 2025; Qi and Zhu, 2018), they often require large, task-specific datasets, extensive online interaction, and careful reward design, which can conflict with the real-time and safety constraints of collaborative industrial cells. Moreover, their learned policies may produce discontinuous or non-deterministic behavior during task switching. In contrast, Dynamic Movement Primitives (DMPs) provide a compact and analytically stable motion representation that guarantees smooth, continuous transitions between motion goals. This property is essential for maintaining safe, predictable, and fluent robot motion in our system, where the robot must adapt instantaneously to updated human intention estimates.

### 2.4 Motion onset and cessation detection

In addition to intention recognition, key challenges in human-robot collaboration include detecting *motion onset and cessation* and ensuring *collision prevention*. Motion onset detection determines when prediction should begin. Hassani et al. (2022) used machine learning for onset recognition in rehabilitation, while surveys highlight electromyography (EMG) as a physiological cue for early detection (Carvalho et al., 2023). Other works fuse IMU and EMG to recognize onset and direction in real time (Tortora et al., 2019), and probabilistic motion models have been applied to jointly recognize and predict human motions (Callens et al., 2020). We focus on a simpler approach by leveraging the knowledge of hand positions to determine when the hand enters or leaves areas of interest.

### 2.5 Our previous work and limitations

In our previous work (Mavsar et al., 2021), we developed LSTM networks capable of classifying observed motions using both RGB-D videos and position sequences obtained via a marker-based tracking system. However, when using RGB-D images, variations in background and camera angles strongly affected predictions, while marker-based systems are costly and impractical in dynamic environments. Yan et al. (2019) addressed this by combining skeleton data from a Kinect depth camera with an LSTM for pose prediction, but they did not exploit detailed RGB information. In contrast, publicly available pose estimation methods (Lugaresi et al., 2019; Fang et al., 2022) can provide 2D landmarks that, when combined with depth data, yield 3D human motion trajectories.

Building on this, we propose LSTM- and transformer-based networks with additional convolutional and pooling layers, inspired by the work of Abdu-Aguye et al. (2020), to capture both local and global temporal features. We compare our models against the transformer network by Pettersson and Falkman (2023), which was most effective in gaze-based intention recognition. While their architecture relies on fixed-size data windows, our dataset consists of variable-length and partial trajectories, enabling real-time prediction from incomplete motion sequences.

## 3 Materials and methods

**FIGURE 2:**
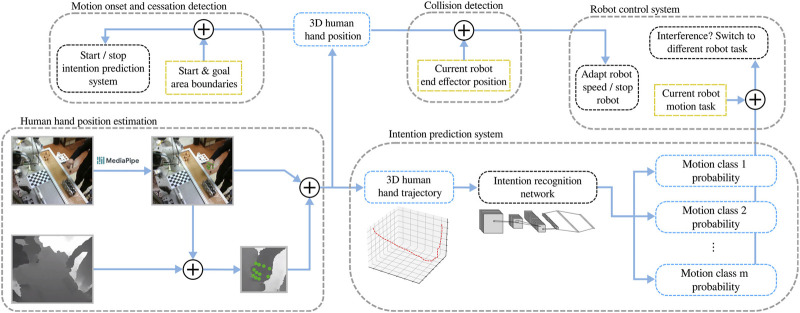
The proposed human-robot collaboration system with intention recognition. The pose of the worker’s hand is passed through a motion classification network to provide predictions of human intention, as well as into a module for collision detection and a module for motion onset and cessation detection. Based on the signal from the motion onset detection module, the intention recognition system is activated and its results are passed into the robot control system to switch to a different robot task if necessary.

In this section we present the methods used to automate the collaborative process, where a human and a robot concurrently perform a task. The proposed approach involves the estimation and prediction of human motion, as well as adaptive robot control that reacts to the actions of the human worker. The diagram of the control system, integrating the proposed methods, is shown in Figure 2.

### 3.1 Hand position estimation

Several open-source frameworks for human motion estimation are available, typically trained on large datasets, which enhances their effectiveness in diverse environments. By leveraging a general human motion estimation system, we can avoid the need to train custom image processing networks for specific applications. One of the most widely used frameworks for human pose estimation is MediaPipe (Lugaresi et al., 2019), which offers high accuracy in tracking body, hand, and face landmarks while maintaining fast processing speeds, even without the use of GPUs. Our approach utilizes MediaPipe’s hand detection solution, which predicts the 2D pixel locations of 21 hand landmarks from an RGB image. For motion classification, it suffices to focus on a single landmark, as our primary goal is to distinguish the destinations of different hand trajectories. We select the landmark at the top of the index finger, as it is typically the most stable part of the hand when holding an object between the index finger and thumb. However, since the output from MediaPipe consists of 2D pixel locations, we additionally use the camera’s depth image to convert these coordinates into 3D positions within the camera’s coordinate system.

Each hand landmark is represented by its corresponding pixel coordinates $u={\left[u,\;v\right]}^{\text{T}}$ , where u∈[0,W−1],v∈[0,H−1] , and W and H denote the width and height of input camera frames $F\left(t\right)\in R^{W\times H\times 3}$ . Our aim is to obtain the world coordinates of the detected hand landmark, $c={\left[x,y,z\right]}^{\text{T}}$ . Let’s denote the landmark position in camera coordinates as $c_{c}={\left[x_{c},y_{c},z_{c}\right]}^{\text{T}}$ . MediaPipe returns the pixel coordinates of each hand landmark. When using a depth camera with aligned color and depth frames, we can take the calculated pixel coordinates of the landmark, u and v , and find the value of the depth image at this location. This value is the distance of the landmark from the origin of the camera coordinate system, i.e., $z_{c}$ . For the calculation of the remaining camera coordinates $x_{c}$ and $y_{c}$ , we start with the relationship between a 3D point c and its image projection u as per Zhang (2000):

$$s\left[\begin{array}{c} u\\ 1 \end{array}\right]=A\left[\begin{array}{cc} R & t \end{array}\right]\left[\begin{array}{c} c\\ 1 \end{array}\right]=Ac_{c}.$$

Here A is the camera intrinsic matrix,

$$A=\left[\begin{array}{ccc} \alpha & \gamma & u_{0}\\ 0 & \beta & v_{0}\\ 0 & 0 & 1 \end{array}\right]$$

with $\left(u_{0},v_{0}\right)$ being the coordinates of the principle point, α and β the scale factors in image’s u and v axes, and γ the skew of image axes. R and t are the extrinsic parameters, denoting rotation and translation of the world coordinate system related to the camera coordinate system.

Writing out the equation system

$$s\left[\begin{array}{c} u\\ v\\ 1 \end{array}\right]=A\left[\begin{array}{c} x_{c}\\ y_{c}\\ z_{c} \end{array}\right],$$

we can compute $x_{c}$ and $y_{c}$ :

$$x_{c}=z_{c}\left(\frac{u-u_{0}}{\alpha }-\gamma \frac{v-v_{0}}{\alpha \beta }\right),$$

$$y_{c}=z_{c}\;\frac{v-v_{0}}{\beta }.$$

In order to obtain the landmark location in the world coordinate system, we use a matrix that defines the transformation from world to camera coordinates:

$$c=R^{\text{T}}c_{c}-R^{\text{T}}t.$$

To compute the intrinsic camera parameters A , we move the calibration board to several different locations within the workcell and gather the calibration data. For the last location, we place the calibration board at the position and orientation coinciding with the origin of the world coordinate system. The intrinsic camera parameters and the transformation matrices from the camera coordinate system to all locations of the calibration board can then be computed using the method described by Zhang (2000). As the location of the last placement of the calibration board coincides with the world coordinate system, it corresponds to the transformation matrix [R,t] from world to camera coordinates.

Using the above procedure, we can sample a sequence of hand positions $c\left(t_{j}\right)\in R^{3},\;\;j=1,\ldots ,n$ , from a camera stream of the observed human worker’s motion. This way we obtain the input data for our system for intention recognition, which is the basis for guiding the robot in a collaborative setting and realizing safe human-robot collaboration.

### 3.2 Intention recognition

We propose a system for classification of the human worker’s motion based on partial hand position trajectories. The output consists of predicted motion classes from a predefined set of possible motions, denoted as k∈{1,…,m} , where m represents the total number of classes. These motion classes correspond to different versions of the collaborative task the worker can perform. In the practical experiment described in Section 4, several distinct slots are available for the human worker to complete an assembly task. The system predicts the specific motion category towards a goal slot where the worker intends to place a workpiece and directs the robot to simultaneously perform the assembly task at a different slot. Although human motions in our experiment are related to the goal slots, the proposed system is adaptable and can classify a wide range of motion classes.

As described in Section 3.1, the RGB image sequences of the observed human motion are processed using MediaPipe and combined with depth images to obtain hand position trajectories $c\left(t\right)\in R^{3}$ . These trajectories are passed to the intention recognition neural network that classifies the observed motion. We compare three different architectures to classify human hand position trajectories, namely a custom LSTM network, a custom transformer-based network, and a transformer-based network proposed by Pettersson and Falkman (2023).

The transformer architecture originally consists of an encoder and a decoder network. For sequence-to-sequence tasks, both the encoder and decoder part of the transformer architecture are used. However, for classification tasks it is customary to utilize only the encoder part of the transformer (Pettersson and Falkman, 2023; Liang et al., 2022), which is also how we design our proposed transformer architecture. Inspired by Abdu-Aguye et al. (2020), the two proposed networks (i.e. LSTM and transformer) include also convolutional and pooling layers. The aim of the convolutional layers is to extract spatial information from input trajectories, while the pooling layers of different sizes retrieve both local and global temporal properties.

Although LSTM and transformer architectures are well-established, our novelty lies in adapting and integrating them for real-time intention recognition from partial and variable-length hand trajectories. We also introduce a lightweight preprocessing module with multi-scale convolutional and pooling layers to extract both local and global temporal features, improving early prediction accuracy from incomplete motion data. Our networks operate continuously at 15 Hz, enabling real-time updates. The proposed models are further integrated with a DMP-based control scheme to achieve smooth, adaptive robot motion, representing the key methodological contribution of this work.

**FIGURE 3:**
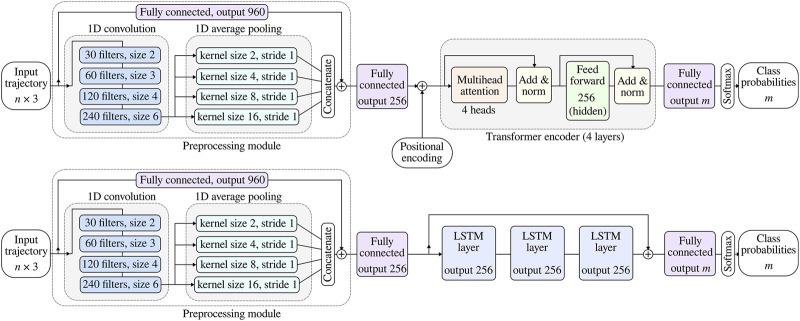
The proposed networks for intention recognition based on input human hand trajectory. The lefthand side of both architectures includes the preprocessing modules with convolutional and pooling layers, which process the input trajectory and pass the output into either transformer-based or LSTM-based network. All convolutional, fully connected and LSTM layers are followed by nonlinear activation layers, which are not shown for simplicity.

The two proposed networks are graphically shown in Figure 3. In both cases, the input is a sampled variable-length hand trajectory $c_{n}={\left[c\left(t_{1}\right),c\left(t_{2}\right),\ldots c\left(t_{n}\right)\right]}^{\text{T}}\in R^{n\times 3}$ . Both networks are trained on partial to full hand trajectories. The input data are first processed by convolutional layers to increase the number of channels, and pooling layers with kernels (or windows) of different sizes and a stride of 1 in order to extract local and global temporal changes. The resulting channels are then concatenated into n vectors of size 960 and added to a signal of size n×960 , obtained by passing the input trajectory through a fully connected layer with 960 neurons, which bypasses the convolutional and pooling layers. This forms a residual connection, which has been shown to ease training and improve performance of neural networks (Quan et al., 2021). Each vector in the resulting sum is finally processed by the same fully connected network with 256 output neurons, giving an overall output of size n×256 . The data processed this way is then fed into either a transformer network or an LSTM network.

When using the transformer (top architecture in Figure 3), the input is first augmented by adding positional encodings, as proposed by Vaswani et al. (2017). Positional encoding injects information about the relative position of individual sequence elements. The result is passed through 4 heads of the multihead attention module with learnable weights. This module allows processing of variable-length sequences and extracts dependencies between different elements in the input sequence, i.e. hand trajectory. Let’s denote the module’s input as $h\in R^{n\times 256}$ . Each element in the input sequence is first passed separately through three different linear networks of each attention module head, followed by a scaled dot product of the three resulting sequences

$$a_{i}=softmax\left(\frac{\left(hW_{q,i}\right){\left(hW_{k,i}\right)}^{T}}{\sqrt{d_{in}}}\right)hW_{v,i},$$

where $d_{in}$ is the dimension of the input samples, in our case 256, and $W_{q,i},W_{k,i},W_{v,i}\in R^{\mathrm{256\times 256}}$ are the weights of linear networks included in the i -th head. The resulting output vectors $a_{i}\in R^{n\times 256},\;\;i=1,\ldots ,4$ , are concatenated into $a\in R^{n\times 1024}$ and multiplied with learnable weights $W_{o}\in R^{\mathrm{1024\times 256}}$ to again obtain output of size n×256 . This is added to the input h and normalized. Each row vector of dimension 256 is then passed through a feed forward network with a hidden dimension of 256, producing the overall output of size n×256 . This is followed by another add and norm operation. The entire process is repeated 4 times, i.e. we use 4 encoder layers. From the transformer output matrix, the vector on the last, i.e. n -th, row is extracted and finally passed through another fully connected network with output layer of size m , where m denotes the number of motion classes. A softmax function is applied to obtain a probability distribution across motion classes.

When the LSTM network (bottom architecture in Figure 3) is used, the output of the preprocessing module is passed through three consecutive LSTM layers with a recurrent structure, which stores information through different time steps. Elements in the input sequence are therefore processed one after the other, with internal LSTM states being updated in each iteration. The output of the final LSTM layer is added to the input into the first LSTM in each time step, forming a residual connection, similar to the one in the preprocessing module. The result is passed through a fully connected network with output layer of size m , and again the softmax function is applied to obtain a probability distribution across motion classes. Note that for an efficient LSTM implementation, the partial input sequences of length n do not need to be passed in their entirety through the LSTM architecture in each time step, since only the last 16 samples change in each time step (due to the use of convolutional filters and average pooling). For this reason, the internal states of all LSTM layers at 16 time steps in the past must be stored so that we compute the new LSTM output using only the latest 16 elements in the input sequence.

The performance of the proposed networks was compared to the best-performing transformer architecture by Pettersson and Falkman (2023) that was used for gaze-based human intention recognition. Original implementation has two parallel output layers, which we merged into one output layer with m outputs, representing motion classes, for use in our experiments. The rest of the architecture was kept the same.

The data used for training of the intention recognition network consists of the following trajectory sample–motion class pairs:

$$D={\left\{{\left\{c_{ij}\right\}}_{i=1}^{L_{j}},\;k_{j}\right\}}_{j=1}^{M},\;c_{ij}\in R^{3},\;k_{j}\in \left\{1,\ldots ,m\right\},$$ (1)

where M is the number of training trajectories and $L_{j}$ denotes the number of samples for the j -th trajectory. To implement the loss function, cross entropy minimization is employed. The networks from Figure 3 output a probability distribution $p=\left\{p_{1},p_{2},\ldots ,p_{m}\right\}$ over m possible classes (versions of the task) by utilizing the softmax layer. Given the predicted probability distribution $p_{n}$ for a partial trajectory with n samples and a correct target class k , the loss is defined as

$$L_{n}\left(p_{n},k\right)=-\log\left(p_{n,k}\right),$$

where $p_{n,k}$ represents the predicted probability of the correct class k when using a partial trajectory with n samples as input to the neural network.

A weighted sum of losses at each sampling step is employed to decrease the significance of early input values and increase the significance of later values. For each input trajectory of length L , the total loss is given as

$$L=\frac{1}{L}\overset{L}{\underset{n=1}{\sum }}\gamma_{n}L_{n}\left(p_{n,k}\right),$$

where $\gamma_{n}$ represents the weight for the n -th input, computed using a logistic function

$$\gamma_{n}=\frac{1}{1+e^{-\alpha \frac{n-1}{L-1}+0.5}}.$$

#### 3.2.1 Detection of motion onset and cessation

Intention recognition processes sequences of hand positions to predict the task currently performed by the human worker. However, predictions should only be made during active motion, which requires determining when a movement begins and ends. To address this, we implemented a motion onset and cessation detection mechanism that automatically activates and deactivates the intention recognition system based on the worker’s hand position. Two 3D regions are defined within the workcell: a starting area, where workpieces are picked up, and a goal area, where they are placed.

Motion onset is detected when the worker’s hand leaves the starting area, marking the beginning of the prediction process. Motion cessation is recognized when the hand enters the goal area, indicating the completion of a movement. To improve detection robustness, a small number of samples before and after each transition are also included in the processing pipeline. This approach enables precise timing of prediction sessions and ensures that the intention recognition operates autonomously. The system continuously tracks goal-slot occupancy based on predicted intentions, allowing the robot to adapt its motion and continue the assembly task at available locations.

### 3.3 Robot trajectory generation and switching

We use Dynamic Movement Primitive (DMP) (Ijspeert et al., 2013) representation specified in Cartesian space (Ude et al., 2014) to specify robot motion in a collaborative task. DMPs are well-suited to represent robot trajectories in HRC environments because they can be used to smoothly pull the robot towards a new motion trajectory when the desired motion changes. This makes it possible to generate a smooth transition when switching from one trajectory to another. By specifying trajectories in Cartesian space, we ensure that transitions are smooth in Cartesian space, which reduces the chance of collisions with the environment, since the switching between DMPs results in predictable trajectories.

In our setup, each of the classes is associated with a specific collaborative robot motion. The proposed neural network architectures in Figure 3 generate a new class prediction after each input sample is processed. As the intention recognition system is not perfect, the predicted class for the observed motion may change as more frames become available. In general, the prediction accuracy improves as more input data points become available. This requires that the robot is capable of smoothly switching from one trajectory to another when the predicted class changes. DMPs are well suited for this purpose.

We adopted the third-order DMP system (Schaal et al., 2005; Nemec and Ude, 2012) for joint space trajectories to Cartesian space robot trajectories. In a Cartesian space DMP, the robot’s motion is specified by its position $y\left(t\right)\in R^{3}$ and orientation trajectory $q\left(t\right)\in R^{4}$ , where q(t) denotes the unit quaternions at time t . In the third-order DMP system, the position trajectory can be described by the following system of differential equations:

$$\tau \dot{v}=K_{p}\left(r-y\right)-D_{p}v-xK_{p}\left(r-y_{0}\right)+K_{p}f_{p}\left(x\right),$$ (2)

$$\tau \dot{y}=v,$$ (3)

$$\tau \dot{r}=H_{p}\left(g_{p}-r\right),$$ (4)

where r , $v\in R^{3}$ are auxiliary variables, $y_{0}$ , $g_{p}\in R^{3}$ are the start and end position, respectively, $K_{p}$ , $H_{p}\in R^{3\times 3}$ are spring matrices, $D_{p}\in R^{3\times 3}$ is a damping matrix, and τ>0 is a temporal scaling factor, usually set equal to the duration of motion. We set $K_{p}=K_{p}I$ , $D_{p}=D_{p}I$ , $H_{p}=H_{p}I$ , $D_{p}=2\sqrt{K_{p}}$ , $H=\sqrt{K_{p}}$ , $K_{p}>0$ , which provides for the critical damping of the dynamic system. The phase variable x is used to remove the direct time dependency from the DMP formulation

$$\tau \dot{x}=-\alpha_{x}x,$$ (5)

where $\alpha_{x}>0$ is a positive constant. The forcing term $f_{p}$ from Equation 2 is defined as a linear combination of M radial basis functions

$$f_{p}\left(x\right)=\frac{1}{\sum_{i=1}^{M}\Psi_{i}\left(x\right)}\overset{M}{\underset{i=1}{\sum }}x\Psi_{i}\left(x\right)w_{i}^{p},\;\Psi_{i}\left(x\right)=\exp\left(-h_{i}{\left(x-c_{i}\right)}^{2}\right),$$ (6)

with weights $w_{i}^{p}\in R^{3}$ set in such a way that by integrating the equation system Equations 2–5, we obtain the desired trajectory y starting at the initial position $y_{0}$ and ending at the goal position $g_{p}$ . See (Ude et al., 2020) for more details about how to compute $w_{i}^{p}$ . The desired robot trajectory is obtained by integrating the differential equation system Equations 2–5, with the initial values set to $y=y_{0}$ , v=0 , r=g , and x=1 . Note that if the goal position $g_{p}$ changes abruptly, r and consequently y converge to the new goal position without causing any discontinuities in the velocity and acceleration of y .

A DMP equation system for standard, i.e., second-order DMPs in a unit quaternion space has been proposed by Ude et al. (2014). Building on this approach, we propose the following equations for third-order quaternion DMPs:

$$\tau \dot{\eta }=K_{o}2\log\left(o*\bar{q}\right)-D_{o}\eta +K_{o}f_{o}\left(x\right),$$ (7)

$$\tau \dot{q}=\frac{1}{2}\eta *q,$$ (8)

$$\tau \omega_{o}=H_{o}2\log\left(g_{o}*\bar{o}\right),$$ (9)

where $\eta \in R^{3}$ and $o\in R^{4}$ are auxiliary variables, and $\omega_{o}\in R^{3}$ is the angular velocity of the auxiliary unit quaternion trajectory o . ∗ denotes the quaternion product and $\bar{q}$ is the conjugate of quaternion q . Note that Equation 7 is not completely analogous to Equation 2. Namely, we have omitted the term $-xK_{o}2\log\left(o*{\bar{q}}_{0}\right)$ because this term causes problems when computing the quaternion DMP’s initial state. Variables $q_{0}$ , $g_{o}\in R^{4}$ are the unit quaternions specifying start and end orientation, respectively. τ is the temporal scaling factor, just like in Equations 2–5. $K_{o}$ , $H_{o}\in R^{3\times 3}$ , $D_{o}\in R^{3\times 3}$ are diagonal positive definite matrices defined similarly as in Equations 2–4. The forcing term $f_{o}$ is defined as $f_{p}$ in Equation 6. Finally, the unit quaternion logarithm is defined as follows

$$\log\left(q\right)=\log\left({\left[v,u^{\text{T}}\right]}^{\text{T}}\right)=\left\{\begin{array}{c} \arccos\left(v\right)\frac{u}{\left\|u\right\|},\;u\neq 0\;\\ {\left[0,0,0\right]}^{\text{T}},\;otherwise\; \end{array}\right.,$$

where v∈R and $u\in R^{3}$ are the scalar and vector part of unit quaternion q .

The differential Equation 5 is used jointly with the position DMP to integrate the phase. The distinguishing property of the third order system Equations 7–9 compared to the standard second-order quaternion DMP (Ude et al., 2014) is that it smoothly transitions the orientation trajectory to a new goal when the goal orientation $g_{o}$ changes.

In our practical experiment, the robot and human worker both start moving towards a slot where they intend to perform the required assembly operation. As explained in Section 3.1, the human worker motion is observed by an RGB-D camera and if the intention recognition system described in Section 3.2 estimates that the human worker’s target slot is the same as the currently selected robot’s target slot, the robot motion is adapted towards a different slot.

Let’s denote the current DMP integration state as $y_{p},v_{p},r_{p}$ and the terms defined by the previous and next DMP (temporal scaling factor, forcing term, end and initial configuration) as $\tau_{p},f_{p},g_{p},y_{0,p}$ and $\tau_{n},f_{n},g_{n},y_{0,n}$ , respectively. To ensure that the position, velocity and acceleration of robot motion remain smooth when switching to a different goal position, we initialize the next DMP integration states $y_{n},v_{n},r_{n}$ as

$$y_{n}=y_{p},$$ (10)

$$v_{n}=\frac{\tau_{n}}{\tau_{p}}v_{p},$$ (11)

$$r_{n}=\frac{\tau_{n}^{2}}{\tau_{p}^{2}}r_{p}+\frac{1-\tau_{n}^{2}/\tau_{p}^{2}}{1-x}y_{p}+\frac{\tau_{p}\tau_{n}-\tau_{n}^{2}}{\tau_{p}^{2}\left(1-x\right)}K^{-1}Dv_{p}+\frac{1}{1-x}\left(\frac{\tau_{n}^{2}}{\tau_{p}^{2}}\left(xy_{0,p}+f_{p}\left(x\right)\right)-xy_{0,n}-f_{n}\left(x\right)\right).$$ (12)

We continue the integration from the current phase x using the DMP parameters of the new trajectory, starting with values Equations 10–12. These initial values are not guaranteed to lie on the initially programmed trajectory. However, since every DMP defines a control policy, the integration converges to the new desired motion.

Switching to a new quaternion DMP occurs in a similar way. For the initialization of the variables of the orientational part of the trajectory, let’s denote the current Cartesian DMP integration state as $q_{p},\eta_{p},o_{p}$ and the terms defined by the current and next Cartesian DMP (temporal scaling factor, forcing term, end and initial orientation) as $\tau_{p},f_{o,p},g_{o,p},q_{0,p}$ and $\tau_{n},f_{o,n},g_{o,n},q_{0,n}$ , respectively. The next DMP integration state $q_{n},\eta_{n},o_{n}$ should be initialized so that the position, angular velocity and angular acceleration of the robot motion remain smooth, i.e., $q_{p}=q_{n},\;\omega_{\text{p}}=\omega_{\text{n}},\;{\dot{\omega }}_{\text{p}}={\dot{\omega }}_{\text{n}}$ . By taking into account that $q_{n}$ is a unit quaternion, we can use Equations 7–9 to compute the following initialization values for the integration of the next quaternion DMP, starting at the current phase x :

$$q_{n}=q_{p},$$

$$\eta_{n}=\frac{\tau_{n}}{\tau_{p}}\eta_{p}$$

$$o_{n}=\exp\left(\left(1-\frac{\tau_{n}}{\tau_{p}}\right)\frac{\tau_{n}D_{o}\eta_{p}}{2\tau_{p}K_{o}}+\right.\left.\frac{\tau_{n}^{2}}{\tau_{p}^{2}}\left(\log\left(o_{p}*\bar{q_{p}}\right)+\frac{f_{o,p}}{2}\right)-\frac{f_{o,n}}{2}\right)*q_{n}.$$

### 3.4 Collision prevention

To prevent the worker and the robot to collide while simultaneously performing a collaborative task, we developed a robot control system that adapts the robot’s speed based on the distance between the end effector and the worker’s hand. This is necessary to prevent interference and injury. By utilizing the RGB-D camera and the procedure from Section 3.1, we obtain the 3D position of the worker’s hand in world coordinates at all times.

We adapt the speed of the robot by changing the τ parameter, used in Equations 2–9 for generation of the robot trajectory. The parameter is adapted based on the distance of the robot end effector to the worker’s hand in such a way, that the speed and thus τ is not changed when the distance is larger than 30 cm and the speed is set to zero when the distance is less than 5 cm, i.e., τ is increased towards infinity. For distances between these two values, a minimum jerk polynomial (Spong et al., 2006) is defined to smoothly increase τ . By denoting the distance between the robot and hand as d=‖y−c‖ , the “safe” distance of 30 cm as $d_{s}$ and “unsafe” distance of 5 cm as $d_{u}$ , we can then write the expression for $k_{\tau }$ , which modifies the parameter τ to obtain the adapted parameter $\tilde{\tau }$ ,

$$\tilde{\tau }=\frac{1}{k_{\tau }\left(d\right)}\tau ,$$

where

$$k_{\tau }\left(d\right)=\left\{\begin{array}{cc} 1,\; & d\geq d_{s},\\ \frac{10{\left(d-d_{u}\right)}^{3}}{{\left(d_{s}-d_{u}\right)}^{3}}-\frac{15{\left(d-d_{u}\right)}^{4}}{{\left(d_{s}-d_{u}\right)}^{4}}+\frac{6{\left(d-d_{u}\right)}^{5}}{{\left(d_{s}-d_{u}\right)}^{5}},\; & d_{s}>d\geq d_{u}+\delta ,\\ \varepsilon .\; & d<d_{u}+\delta , \end{array}\right.$$

where $\delta =10^{-8}$ and $\varepsilon =k_{\tau }\left(d_{u}+\delta \right)$ . As $k_{\tau }$ approaches 0, $\tilde{\tau }$ increases towards infinity. We set the value of $k_{\tau }$ for $d\leq d_{u}+\delta$ to ε to prevent division by zero. The coefficients for the minimum jerk polynomial, which is a function of d , were calculated by considering the boundary conditions, i.e., the first and second derivatives of the polynomial at $d=d_{u}$ and $d=d_{s}$ are zero, while $k_{\tau }\left(d_{u}\right)=0,\;k_{\tau }\left(d_{s}\right)=1$ . This way we ensure the smoothness of robot motion even when τ starts changing.

## 4 Experiments and results

We aimed to evaluate the performance of our proposed intention recognition system and to test the integrated HRC supervision system in a real use case scenario. We compared several different architectures for human hand trajectory classification, namely a transformer-based network by Pettersson and Falkman (2023) and our two proposed LSTM and transformer networks. The networks were used to detect the goal slot where the human worker intended to place an object. The best performing network was tested in a real-life industrial scenario, along with the collision prevention system and the motion onset and cessation detection system.

### 4.1 Experimental setup

The experimental setting is presented in Figure 1. The setup is the same as in a real-life industrial scenario for the production of car starters. The robot and the human perform the same task, i.e., they pick up a copper ring from the loading area and insert it into the casting model on the table. They execute the task simultaneously, which means that the human may attempt to insert a ring into the same slot as the robot.

The casting model is composed of four insertion slots, where the robot and the human can access all slots. The aim of the intention recognition system is to quickly predict the slot where the human operator intends to place the copper ring and adapt the robot motion plan to prevent interference in the workspace.

#### 4.1.1 Data acquisition

During the process of data gathering, the human worker was given instructions to move an object from the designated starting area and place it into one of the four available slots of the casting model on the table. The subjects performed various motions, replicating the actions typically carried out in production environments, where workers execute smooth and deliberate movements.

**FIGURE 4:**
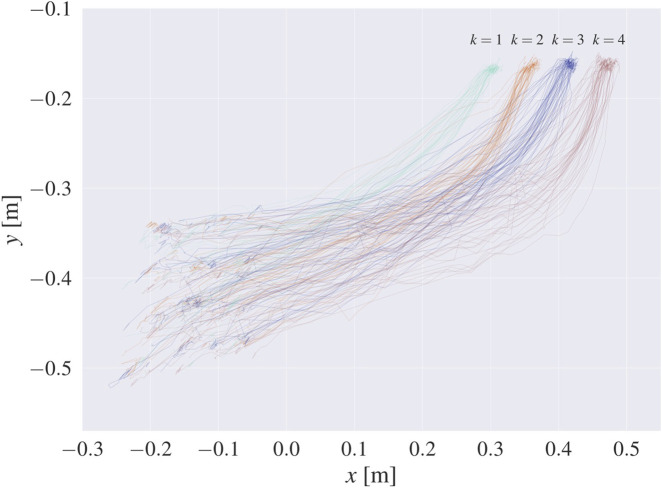
Raw recorded trajectories shown in the *xy*-plane in world coordinates, with different colors denoting different goal slots (k∈{1,…,4}) . The motions begin on the bottom left side of the graph and end on the upper right side, where the four different goal areas are clearly distinguishable.

At the initiation of each subject’s motion, video recording was started at a rate of 30 Hz, using the Intel RealSense Depth Camera D435. The recording ceased when the subject reached one of the slots on the casting model. This procedure resulted in 215 samples, consisting of RGB-D videos and task version labels k∈{1,…,4} , indicating the slot where the object was placed (recorded trajectories are shown in Figure 4). By employing the techniques described in Section 3.1, we transformed the RGB-D videos into 3D trajectories of hand motion c(t) , finally obtaining a dataset from Equation 1.

To evaluate the performance of our networks, we randomly split the available data five times into non-overlapping training, validation, and testing subsets. The testing subsets between the splits are non-overlapping. The division of data into different splits allows us to evaluate statistical significance of our results. After each subdivision, we digitally enlarged our training, validation, and testing subsets by randomizing the available data (trajectories). As explained above, we employ DMPs to specify the desired robot motions, but their properties allow us to utilize them for data randomization as well, since they result in a smooth and natural motion even if random noise is introduced in their parameters. Thus for digital data augmentation, we encoded the recorded trajectories using DMPs and randomly changed the initial position $y_{0}$ , final position g and trajectory duration τ , where the scale of noise was set based on the variation of these parameters in the training data. We then integrated Equations 2–5 to obtain modified trajectories and finally introduced random Gaussian noise to hand position measurements. This resulted in a dataset with higher variability, encompassing a wide range of subjects’ motions. The total number of training, validation and test trajectories obtained in this way was 890, 300 and 235, respectively, for each data split.

#### 4.1.2 Training method

We train all networks using trajectories in the training dataset D from Equation 1, including partial trajectories. This approach enables the network to learn and predict not only from complete trajectories but also from partial observations, which is crucial in real-time HRC scenarios, where the system needs to make predictions based on incomplete or partial data.

The proposed networks were implemented using the PyTorch (Paszke et al., 2019) framework and trained using the Adam optimization algorithm (Kingma and Ba, 2015) with a learning rate of 0.001 and a batch size of 40, where the training was stopped after 100 consecutive epochs of no error reduction on the validation set.

### 4.2 Results

The intention recognition networks were evaluated on five different datasets, obtained through the process described in Section 4.1.1. The input samples (sequences of hand position measurements c ) were passed through the proposed networks to obtain the predicted intention of the human worker, i.e. the label of the target slot the worker is moving the object towards.

Upon processing each element of the input sample, the networks compute the probability distributions across four target slots. These distributions represent the predicted probabilities for each slot. As new position measurements (calculated by processing camera frames) are received, the predicted probabilities are continuously updated. This enables the prediction of the worker’s intention in real-time as the motion is being performed.

**FIGURE 5:**
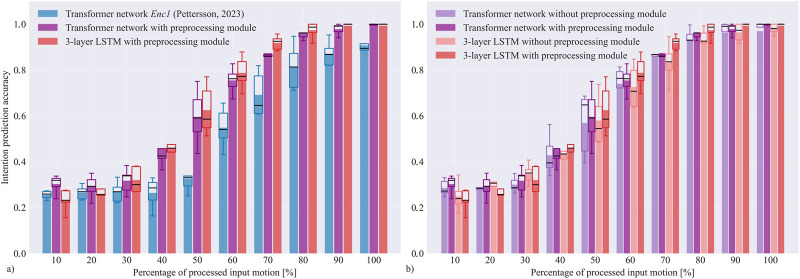
Temporal accuracy of the tested motion classification networks. The left graph **(a)** shows accuracy comparison between the adapted transformer network from (Pettersson and Falkman, 2023) and our proposed LSTM and transformer networks with the preprocessing module (comprising convolutional and pooling layers), while the right graph **(b)** demonstrates the performance of our networks with and without convolution and pooling layers. The presented results were calculated after partial observations of input trajectories, from 10% to 100% . The bars show the mean accuracy across all five data splits for each network at a certain percentage of processed input motion. The box-and-whisker plots display the variation of results across data splits. Boxes show the range of data between the first quartile Q1 (25%) and the third quartile Q3 (75%), black line is the median, and the whiskers extend from the 5-th to the 95-th percentile.

To evaluate and compare the accuracy of intention recognition architectures, we calculated prediction accuracy for all three networks in relation to the percentage of the input motion processed, as shown in Figure 5a. This was done for all 5 data splits, and box-and-whiskers plots were generated to show variability of results across splits. For all tested networks, the results demonstrate that as a larger portion of input motion becomes available, the average accuracy of intention recognition improves. The accuracy is above 90% at the end of motion. For the best performing network, the average accuracy reaches approximately 46% at 40% of motion completion, 92% at 70% of motion and improves to nearly 100% at the end of motion. The network with LSTM layers in average performed best at human motion classification in our experiments, especially towards the end of motion, although its structure is significantly simpler than transformer architecture. This may be due to the dataset being relatively small, while the advantages of transformer networks typically become more pronounced with extremely large datasets (Xu et al., 2021; Wang et al., 2022). Both of our proposed architectures performed significantly better than the transformer-based network by Pettersson and Falkman (2023), with the accuracy being around 15% to 30% higher in the middle part of motion. At 70% and 100% of the processed motion, the average accuracy of the LSTM network is 23% and 9% higher than the network by Pettersson and Falkman (2023), respectively.

**FIGURE 6:**
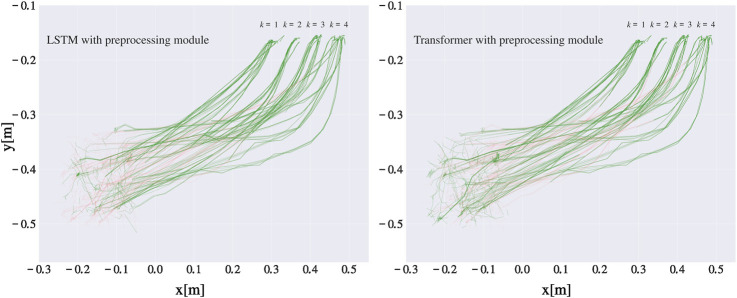
Classification accuracy of the networks on one of the test datasets, shown in the *xy*-plane. Motions start in the left lower part of the graph and end in the right upper part of the graph at one of the four goal slots. Parts of the trajectories where the networks’ predictions were correct are shown in green, while the trajectory parts where the predictions were incorrect are shown in red.

To show the prediction accuracy of the best performing network also in terms of Cartesian distance to the goal slots, we plotted all test trajectories from data split number 1 in the *xy*-plane, and highlighted the parts of trajectories where the network’s classification was correct with green color (see Figure 6), and the parts where classification was wrong with red color. This gives a better overview of how far from the goal slots the networks first give a correct prediction. As expected, the percentage of green, i.e. correctly classified trajectories, increases the closer the worker’s hand is to the goal slots. We can notice that there are slightly more green parts closer to goal slots when LSTM network is used for classification, which is in line with the results from Figure 5. Some trajectories are green from the beginning, more so when the transformer is employed, however this is most probably due to a lucky guess since the available information at the start of motion is minimal.

### 4.3 Ablation study

The two types of the proposed network architectures, i.e. the LSTM and the transformer network, were tested with and without convolutional and pooling layers (preprocessing module) to assess their effect. The accuracy of the resulting networks across five data splits are displayed in Figure 5b.

The positive effect of the preprocessing module is noticeable both with the LSTM architecture, as well as with the transformer network. The average accuracy of motion classification is higher when the preprocessing module is included regardless of the percentage of the processed motion, except at the beginning of motions where limited information is available and the correct predictions are mostly the result of chance. Convolutional and pooling layers improve the accuracy of the LSTM architecture by around 3−9% , with the highest improvement in the middle part of the observed motion. The increase is slightly lower with the transformer, possibly because transformers process entire input sequences in each time step, and may therefore benefit less from preprocessing. However, even a small increase in prediction accuracy can prove important when optimizing HRC tasks, especially towards the end of motion where predictions should be as accurate as possible.

### 4.4 Implementation of a human-robot collaborative task

The proposed intention recognition system was deployed in real-time to control a robot in a collaborative task. In the scenario, a human and a Franka Emika robot collaborated to simultaneously transfer copper rings to one of the available target slots. A video demonstrating the combined methods in the HRC use case is available as Supplementary Material for this paper.

Robot Operating System (ROS) was used to enable communication between the Intel RealSense camera, LSTM network and the Franka Emika robot. The robot was controlled in Cartesian impedance mode, which ensures that it is compliant and yields in the case of collisions, reducing impact forces. Four robot trajectories to each target slot were first recorded using kinesthetic guiding and encoded with DMP parameters (see Section 3.3). During collaborative task execution, the worker’s motion was continuously observed by an RGB-D camera and the frames were processed by MediaPipe to obtain a 3D trajectory of hand motion (see Section 3.1). This trajectory was passed through the LSTM architecture to obtain the predicted probability distribution across target slots Ψ , which was then sent to the robot control system. Slot k with the highest probability was selected as the intended target of the worker’s motion and the robot reacted accordingly; if the predicted worker’s target slot was the same or adjacent to the goal slot of the robot, the robot would change the goal slot to the farthest one possible (e.g., if the robot was moving towards slot k=2 , and the worker’s intention was predicted as k=1 , the robot would switch to slot k=4 ). The adjustment of robot motion in response to new predictions was implemented by switching from one DMP to another (as described in Section 3.3).

**FIGURE 7:**
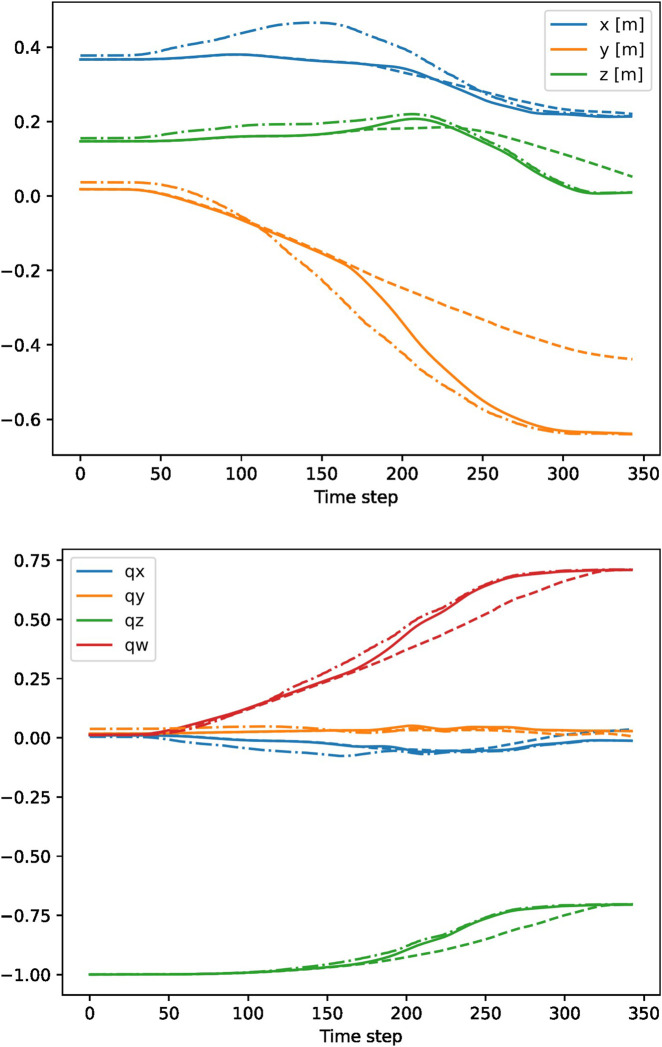
Example switching between two DMPs for positional (top) and quaternion (bottom) part of the trajectory. The robot switched from a motion towards slot k=1 (dashed lines) to a motion towards slot k=4 (dash-dotted lines). The executed motion is shown with solid lines. The robot was controlled with a frequency of 100 Hz.

The HRC supervision system proved to enhance the efficiency of human-robot cooperation during the task, since it quickly adapted the robot motion to prevent placing an object into the same slot as the human, which resulted in a robust collaborative task execution. The overall safety was also improved, as the robot slowed down or stopped if the worker’s hand came too close to the robot end effector. The predictions were output at a rate of 15 Hz, showing that real-time performance can be achieved. Note that the robot is controlled with a much higher frequency (1 kHz). The effectiveness of the proposed DMP-based control system is demonstrated in Figure 7. We can see that the robot can smoothly switch from one motion to another without abrupt changes.

## 5 Discussion

In this paper we propose an integrated system for supervision and control of a human-robot collaboration task. We combine several techniques for ensuring a safe and dynamic cooperation between a human worker and a robot, such as predicting the worker’s intention, detecting the position of the worker’s hand to prevent collisions, and automatic motion onset and cessation detection.

To implement the worker’s intention recognition, we compared three different approaches to classify the human’s hand trajectory. We trained two custom architectures with convolutional and pooling layers followed by LSTM or transformer layers, and an existing transformer-based architecture. Both of our networks performed significantly better than the existing one, with LSTM-based network performing slightly better than the transformer-based network. Although their structure is more complex and allows for powerful sequence processing, transformers typically require large amounts of training data to perform well (Xu et al., 2021; Wang et al., 2022), while data is rather limited in our use case. Another reason for LSTM’s better performance may be the nature of the input data. We process continuous motion trajectories, meaning that the output should not change abruptly when new measurements are processed. LSTM networks inherently take this into account by iteratively adapting the hidden states with each new input, reducing the chance of abrupt changes, while transformers compute attention over the entire input sequence each time. We also showed that the added preprocessing module positively contributes to the performance of both the LSTM network and the transformer network. The increase in accuracy is slightly higher with the LSTM network, which may be due to the fact that transformers process entire input sequences, thus obtaining less additional information from convolution and pooling. The methods were applied to a relatively simple task, i.e., four different classes, however they can be easily extended to a more complex problem by increasing the output layer size.

The obtained results show that a transformer network, while more complex, may not be fit for all tasks, especially where only a limited amount of training data is available. One advantage of LSTMs over transformers that is especially important for real-time processing is also that the sensor data at each time step can be fed continuously into the LSTM to obtain a new output state, while entire partial sequences of input data must be fed into the transformer network at each time step.

Another important contribution of this paper is the third-order quaternion based DMP representation, which allows for smooth switching up to the second order derivatives. This is important for ensuring smooth robot motion when the intended goal position and orientation change. We demonstrated the effectiveness of the proposed HRC system in a real industrial scenario. The system was shown to improve safety and fluency of human-robot collaboration due to better robot task selection and interference avoidance.

The proposed framework currently applies to tasks where the robot has prior knowledge of all possible goals and trajectories, which are acquired through the programming by demonstration. This is usually the case in most practical situations in industrial environments. While this ensures robustness and safety in structured collaborative tasks, it limits flexibility in more dynamic environments. Additional supporting systems would be needed for the robot to fully exploit the estimation of human movement to re-plan its movements and goals in real time.

For future work we plan to address a potential issue that can occur when the robot blocks the view of the worker’s hand, disabling hand position estimation, and, consequently, intention recognition. This could be prevented by including multiple cameras in the workcell to ensure that the worker’s hand is always in line of sight. Another improvement of the system would be for the robot to smoothly avoid the worker’s arm without significantly reducing speed. In the future, we also plan to test transformer architectures that are optimized for smaller datasets, with some solutions proposed by Xu et al. (2021) and Wang et al. (2022).

## Data Availability

The raw data supporting the conclusions of this article will be made available by the authors, without undue reservation.
